# Predictors of Acute Respiratory Distress Syndrome in Patients with Paraquat Intoxication

**DOI:** 10.1371/journal.pone.0082695

**Published:** 2013-12-11

**Authors:** Cheng-Hao Weng, Ching-Chih Hu, Ja-Liang Lin, Dan-Tzu Lin-Tan, Ching-Wei Hsu, Tzung-Hai Yen

**Affiliations:** 1 Department of Nephrology, Chang Gung Memorial Hospital, Linkou Medical Center, Taoyuan, Taiwan; 2 Chang Gung University, College of Medicine, Taoyuan, Taiwan; 3 Department of Hepatogastroenterology and Liver Research Unit, Chang Gung Memorial Hospital, Keelung, Taiwan; Kaohsiung Chang Gung Memorial Hospital, Taiwan

## Abstract

**Introduction:**

Paraquat poisoning is characterized by acute lung injury, pulmonary fibrosis, respiratory failure, and multi-organ failure, resulting in a high rate of mortality and morbidity. The objectives of this study were to identify predictors of acute respiratory distress syndrome (ARDS) in cases of paraquat poisoning and determine the association between these parameters.

**Materials and Methods:**

In total, 187 patients were referred for management of intentional paraquat ingestion between 2000 and 2010. Demographic, clinical, and laboratory data were recorded. Sequential organ failure assessment (SOFA) and Acute Kidney Injury Network (AKIN) scores were collected, and predictors of ARDS were analyzed.

**Results:**

The overall mortality rate for the entire population was 54% (101/187). Furthermore, the mortality rate was higher in the ARDS patients than in the non-ARDS patients (80% vs. 43.80%, *P*<0.001). Additionally, the ARDS patients not only had higher AKIN_48-h_ scores (*P*<0.009), SOFA_48-h_ scores (*P*<0.001), and time to ARDS/nadir PaO_2_ (*P*=0.008) but also suffered from lower nadir PaO_2_ (*P*<0.001), nadir AaDO_2_ (*P*<0.001), and nadir eGFR (*P*=0.001) compared to those in the non-ARDS patients. Moreover, pneumomediastinum episodes were more frequent in the ARDS patients than in the non-ARDS patients (*P*<0.001). A multivariate Cox regression model revealed that blood paraquat concentrations (*P*<0.001), SOFA_48-h_ scores (*P*=0.001), and steroid and cyclophosphamide pulse therapies (*P*=0.024) were significant predictors of ARDS. The cumulative survival rates differed significantly (*P*<0.001) between patients with SOFA_48-h_ scores <3 and SOFA_48-h_ scores ≥3, with a sensitivity of 95.8%, specificity of 58.4%, and overall correctness of 67.6%. Finally, the area under the receiver operating characteristic (AUROC) analysis showed that SOFA_48-h_ scores (*P*<0.001) had a better discriminatory power than blood paraquat concentrations (*P*=0.01) for predicting ARDS.

**Conclusions:**

The analytical results indicate that SOFA_48-h_ scores, blood paraquat concentrations, and steroid and cyclophosphamide pulse therapies are significantly associated with ARDS complications after paraquat intoxication.

## Introduction

Because pesticides[1] and herbicides[2] are easily accessible, they are frequently ingested in Taiwan, both intentionally and accidentally. Paraquat is a popular bipyridyl herbicide with a good safety record when used properly. However, the high toxicity of this compound leads to a mortality rate of 60-80%. After ingestion of approximately 40 mL of a 24% paraquat solution, patients generally die within several hours to days from multiple organ failure. After ingesting approximately 16 mL, patients experience moderate to severe poisoning and die within 1–2 weeks from pulmonary fibrosis and severe hypoxemia[3,4]. Many treatment modalities have been developed for paraquat poisoning, including adsorbents, hypo-oxygenation, lung radiotherapy, prolonged extracorporeal detoxification, and lung transplantation. Nevertheless, the usefulness of these approaches remains indeterminate. 

At our hospital, all patients with paraquat intoxication are routinely treated using a standard detoxification protocol[4-8]. This protocol consists of repeated pulses of methylprednisolone and cyclophosphamide, followed by prolonged dexamethasone therapy. With this strategy, it was demonstrated that both respiratory function and blood oxygen concentrations in most patients returned to near-normal in 3–6 months[9]. Methylprednisolone, cyclophosphamide, and dexamethasone are strong anti-inflammatory medical therapies. Therefore, severe pulmonary inflammation which eventually will progress to pulmonary fibrosis, plays an essential role in producing lethal hypoxemia after paraquat poisoning. Nevertheless, many of our patients still die of pulmonary fibrosis and severe hypoxemia within 1–2 weeks of paraquat intoxication. A previous study [2] demonstrated that PaO_2_ (partial pressure of oxygen in arterial blood) and sequential organ failure assessment (SOFA) scores at 48 hours after admission (SOFA_48-h_) were associated with mortality in these patients with paraquat intoxication.

Many patients with paraquat intoxication developed acute respiratory distress syndrome (ARDS), defined as an acute onset of bilateral pulmonary infiltrates, a ratio of PaO_2_ to fraction of inspired oxygen (FiO_2_) of ≤200 mmHg, and pulmonary artery occlusion pressure of ≤18 mmHg or absence of left atrial hypertension[10]. Notably, patients with ARDS often have high incidences of mortality and morbidity.

Data on the clinical predictors of ARDS after paraquat ingestion are lacking in the literature. Paraquat toxicity has been questioned and discussed for decades among international and national regulatory bodies, and non-governmental organizations and many countries have banned its commercial use[11]. This may be the reason why few studies on this issue are published. Therefore, this study attempted to examine the values of SOFA score, Acute Kidney Injury Network (AKIN) score, and other clinical variables in predicting ARDS. As previously mentioned, the SOFA score has been used to predict the mortality of paraquat poisoned patients[2], and it has several advantages: the measurement of PaO_2_/FiO_2_ is not limited to mechanically ventilated patients; calculation of the SOFA score is simpler than calculation of the Acute Physiology and Chronic Health Evaluation (APACHE II) score (which is complex and not suitable for typical hospital inpatients); and it involves only PaO_2_/FiO_2_, platelet count, serum bilirubin concentration, hypotension, Glasgow coma score, and serum creatinine or urine output[2]. In addition, the SOFA score assesses the hepatic toxicity of paraquat[12] , and this is exceptionally important in view of the cross talk between the liver and pulmonary damage[13,14]. In theory, SOFA scores might be good predictors of ARDS in patients with paraquat poisoning. Finally, the reason for including the AKIN score is another similar cross talk between renal and pulmonary damage[15]. At high doses, paraquat can cause acute tubular necrosis, leading to oliguric or nonoliguric renal failure. As a result, renal excretion of paraquat is markedly reduced, resulting in higher serum concentrations and increases in paraquat accumulation in organs, such as the lung and liver. Although, renal damage is reversible if patients have ingested < 40 mg/kg paraquat[16], death may still occur from delayed pulmonary fibrosis and hypoxemia. Therefore, it would be worthy to examine the value of the AKIN score in predicting ARDS after paraquat intoxication.

## Materials and Methods

### Ethics statement

This retrospective observational study complied with the guidelines of the Declaration of Helsinki and was approved by the Medical Ethics Committee of Chang Gung Memorial Hospital, a tertiary referral center located in the northern part of Taiwan. Since this study involved retrospective review of existing data, approval from the Institutional Review Board was obtained, but without specific informed consent from patients[1]. However, informed consent regarding risks associated with acute paraquat poisoning and all treatment modalities (particularly charcoal hemoperfusion) was obtained from all patients upon their initial admission. Furthermore, not only were all data securely protected (by delinking identifying information from the main data sets) and made available only to investigators, but they were also analyzed anonymously. The Institutional Review Board of Chang Gung Memorial Hospital specifically waived the need for consent for these studies. Finally, all primary data were collected according to procedures outlined in epidemiology guidelines that strengthen the reporting of observational studies.

### Patients

In total, 187 patients were referred for management of intentional paraquat ingestion between January 2000 and December 2010. 

### Diagnosis of paraquat poisoning

A presumptive diagnosis of paraquat poisoning was based on exposure history, clinical effects, and physical and laboratory examinations, especially the urine sodium dithionite screening test[4-8]. The urine sodium dithionite reaction depended on the reduction of paraquat by sodium thionite under alkaline conditions to form stable, blue-colored radical ions. The generation of a strong navy or dark blue color generally indicates significant paraquat ingestion and often forebodes a poor prognosis. The urine test was used as a paraquat screen, and a confirmatory diagnosis of paraquat poisoning was only possible by checking blood paraquat concentrations (spectrophotometry, Hitachi, Tokyo, Japan).

### Definition of ARDS

ARDS was defined according to the American-European consensus conference as an acute onset of bilateral pulmonary infiltrates, a ratio of PaO_2_ to fraction of inspired oxygen (FiO_2_) of ≤200 mmHg, and pulmonary artery occlusion pressure of ≤18 mmHg or absence of left atrial hypertension[10].

### Inclusion and exclusion criteria

Patients were included in this study if they were >18 years of age and had urine paraquat tests that showed dark or navy blue coloring (>5 ppm). Patients were excluded from the study if the paraquat exposure was limited to dermal or intravascular exposure[17,18]. Patients were also excluded if they did not have detectable paraquat levels in their urine and blood or if they had major comorbidities, such as cancer or heart, lung, renal, or liver diseases. The diagnoses of major comorbidities were based on detailed clinical, physical, and laboratory examinations. Patients with pre-existing serum creatinine concentrations >1.4 mg/dL, alanine aminotransferase (ALT) concentrations >36 mg/dL, or total bilirubin concentrations >3 mg/dL were also excluded.

### SOFA and AKIN scores (Table 1 and 2)

**Table 1:** SOFA scoring system.

	0	1	2	3	4
PaO_2_/FiO_2_	>400	301–400	201–300	101–200 with respiratory support	≤ 100 with respiratory support
Platelets (1000/µL)	>150	101–150	51–100	21–50	≤20
Bilirubin (mg/dL)	<1.2	1.2–1.9	2.0–5.9	6.0–11.9	>12.0
Hypotension	MAP ≥ 70 mmHg	MAP < 70 mmHg	Dopamine 5 or dobutamine (any dose)*	Dopamine >5 or epi ≤ 0.1 or norepi ≤ 0.1*	Dopamine >15 or epi >0.1 or norepi >0.1*
GCS	15	13–14	10–12	6–9	<6
Cr (mg/dL) or UO	<1.2	1.2–1.9	2.0–3.4	3.5–4.9 or <500 mL/d	>5.0 or <200 mL/d

^*^ Adrenergic agents administered for at least 1 h (doses are given in µg/kg per minute). Abbreviations. PaO_2_: partial pressure of oxygen in arterial blood, FiO_2_: fractional inspired oxygen, MAP: mean arterial pressure, epi: epinephrine, norepi: norepinephrine, GCS: Glasgow Coma Scale score, Cr: creatinine, UO: urine output

**Table 2:** AKIN scoring system.

Category	Serum Cr criteria	Urine output criteria
Stage 1	Increase in serum Cr of ≥0.3 mg/dL or increase to ≥150% to 200% (1.5 to 2-fold) from baseline	< 0.5 mL/kg/h for more than 6 h
Stage 2	Increase in serum Cr to >200% to 300% (>2 to 3-fold) from baseline	< 0.5 mL/kg/h for more than 12 h
Stage 3	Increase in serum Cr to >300% (3-fold) from baseline (or serum Cr of ≥4.0 mg/dL with an acute increase of at least 0.5 mg/dL	< 0.3 mL/kg/h for 24 h or anuria more for 12 h

Cr: creatinine

The following data were collected: baseline demographics, SOFA and AKIN scores 48 hours after admission (SOFA_48-h_ and AKIN_48-h_), and time to ARDS (ARDS patients) or nadir PaO_2_ (non-ARDS patients). The SOFA score consists of 6 variables, each representing an organ system. Each organ system is assigned a point value from 0 (normal) to 4 (high degree of dysfunction/failure). The AKIN criteria classify acute kidney injury into 3 stages of severity (stages 1, 2, and 3)[2].

### Protocol for paraquat detoxification

The paraquat detoxification protocol[4-8] includes gastric lavage with a large amount of 0.9% saline followed by 1 g/kg activated charcoal and 250 mL magnesium citrate through a nasogastric tube. Charcoal haemoperfusion with a charcoal-containing (Adsorba, Gambro, Germany) dialysis machine (Surdial, Nipro, Japan) was initiated if the urine paraquat was >5 ppm. A second session of haemoperfusion was arranged if the urine paraquat was >5 ppm at 4 h after the first haemoperfusion. The protocol also included pulse therapies of cyclophosphamide (15 mg/kg/day) for 2 days as well as methylprednisolone (1 g/day) for 3 days. Intravenous dexamethasone (20 mg/day) was administrated for another 11 days after methylprednisolone pulse therapy. Pulse therapies with cyclophosphamide and methylprednisolone were repeated if the PaO_2_ was <60 mmHg and the duration was >2 weeks after the initial treatment, unless patients had leucopenia (white cell counts < 3000/m^3^). Finally, all patients received normal inspired oxygen therapy (FiO_2_ 21%) throughout their hospitalization.

### Statistical analysis

Data were expressed as mean ± standard deviation or number and percentage in parentheses, unless otherwise stated. All variables were tested for normal distribution using the Kolmogorov–Smirnov test. The Student’s *t* test was used to compare the means of continuous variables and normally distributed data. Otherwise, the Mann–Whitney *U* test was used for non-normally distributed data. Categorical data were analyzed using the chi-square test. Finally, risk factors were assessed by performing univariate Cox regression analysis, and variables that were statistically significant (*P*<0.05) were included in a multivariate analysis by applying a multiple Cox regression based on forward elimination of data. Calibration was assessed using the Hosmer–Lemeshow goodness-of-fit test to compare the number of observed and predicted ARDS in risk groups for the entire range of ARDS probabilities. Discrimination was assessed with the area under the receiver operating characteristic (AUROC) analysis. The AUROCs were compared using a non-parametric approach. AUROC analyses were also used to calculate cutoff values, sensitivity, specificity, and overall correctness. Finally, the cutoff points were calculated by acquiring the best Youden index (sensitivity + specificity - 1). The cumulative survival curves as a function of time were generated using the Kaplan–Meier approach and compared by log-rank test. All statistical tests were 2-tailed, with *P* values <0.05 being considered statistically significant. Data were analyzed using SPSS 12.0 software for Windows (SPSS, Inc., Chicago, IL).

## Results

### Subject characteristics

As shown in Table 3, the patients with paraquat intoxication were 42.1 ± 15.4 years old and were mostly male (77%). The overall hospital mortality for the entire population was 54% (101/187). Furthermore, the ARDS patients demonstrated a higher mortality rate than that for the non-ARDS patients (80% vs. 43.80%, *P*<0.001). Additionally, the ARDS patients not only had higher AKIN_48-h_ scores (*P*<0.009), SOFA_48-h_ scores (*P*<0.001), and time to ARDS/nadir PaO_2_ (*P*=0.008) but also suffered from lower nadir PaO_2_ (*P*<0.001), nadir AaDO_2_ (*P*<0.001), and nadir eGFR (*P*=0.001) compared to those in the non-ARDS patients. Finally, there were more episodes of pneumomediastinum in the ARDS patients than in the non-ARDS patients (*P*<0.001). 

**Table 3:** Baseline demographics and clinical characteristics of paraquat patients with and without ARDS (n=187).

Parameter	All patients (n=187)	ARDS patients (n=50)	Non-ARDS patients (n=137)	*P* value
Age	42.1±15.40	40.56±15.18	42.58±15.60	0.439
Gender (male/female)	144/43	38/12	106/31	1.000
Mortality n (%)	100 (53.48)	40 (80)	60 (43.80)	<0.001***
Hemoperfusion n (%)	167 (89.30)	45 (90)	122 (89.05)	0.785
Time to hospitalization (hours)	13.49±21.13	11.45±20.29	14.22±21.57	0.438
Estimated ingestion amount (mL)	80.91±104.68	92.81±90.411	77.11±109.90	0.375
Blood paraquat concentration (ppm)	4.77±5.61	5.80±6.46	4.32±5.13	0.110
Urine paraquat concentration (ppm)	37.78±18.70	41.68±15.57	36.23±19.62	0.055
Peak AST concentration (U/L)	125.06±139.20	101.40±71.95	136.33±162.24	0.523
Peak ALT concentration (U/L)	183.20±121.03	232.54±258.63	160.29±135.74	0.100
Peak bilirubin concentration (mg/dL)	3.17±1.69	3.58±4.18	2.61±3.21	0.388
AKIN _48-h_ (stage 0/1/2/3)	98/49/17/23	20/11/7/12	78/38/10/11	0.009**
SOFA _48-h_	3.15±2.3	5.10±2.16	2.47±1.96	<0.001**
Time to ARDS (ARDS patients) or time to nadir PaO_2_ (Non-ARDS paients) (days)	7.40±8.02	10.04±9.01	6.47±7.52	0.008**
nadir PaO_2_	61.28±24.99	30.21±7.60	72.16±19.10	<0.001***
nadir AaDO_2_	46.62±28.34	75.03±26.03	36.66±21.90	<0.001***
nadir eGFR	57.05±33.38	43.52±21.47	62.12±35.49	0.001**
Pneumomediastinum (n) (%)	7 (3.7%)	5 (10%)	2 (1.5%)	<0.001***
Pneumothorax (n) (%)	1 (0.5%)	0	1 (2%)	

Note: AaDO_2_ alveolar-arterial oxygen gradient, AKIN Acute Kidney Injury Network, ALT alanine aminotransferase, ARDS acute respiratory distress syndrome, AST aspartate aminotransferase, eGFR estimated glomerular filtration rate, PaO_2_ partial pressure of oxygen in arterial blood, SOFA Sequential Organ Failure Assessment, *P<0.05, **P<0.01, ***P<0.001

### Calibration, discrimination, and correlation for the SOFA scoring system and blood paraquat concentrations

Calibration of SOFA_48-h_ scores was carried out as follows: Hosmer–Lemeshow; *X* ^*2*^_2_ = 6.818, *P* = 0.235 (Table 4). The blood paraquat concentrations had good calibration, as estimated by the Hosmer–Lemeshow goodness-of-fit test. Table 4 shows the goodness of fit for the predicted mortality risk, and the predictive accuracy of SOFA_48-h_ and the blood paraquat concentration. Table 4 lists the discrimination power of SOFA_48-h_ and the blood paraquat concentration. In terms of ARDS prediction (Table 4), the AUROC analysis showed that SOFA_48-h_ scores (*P*<0.001) had a better discriminatory power than blood paraquat concentrations (*P*=0.01).

**Table 4:** Comparison of calibration and discrimination power of SOFA scoring methods for predicting ARDS (n=187).

	Calibration			Discrimination		
	Hosmer-Lemeshow goodness of fit	*df*	*P*	AUROC±SE	95% CI	*P*
Blood paraquat concen- tration (PPM)	8.463	7	0.294	0.622±0.045	0.534-0.711	0.01*
SOFA_48-h_	6.818	5	0.235	0.827±0.030	0.769-0.886	<0.001***

Note: AUROC area under receiver operating characteristic CI confidence interval, *df* degree of freedom, SE standard error, SOFA sequential organ failure assessment, *P<0.05, ***P<0.001

### Clinical predictors of ARDS

**Figure 1:**
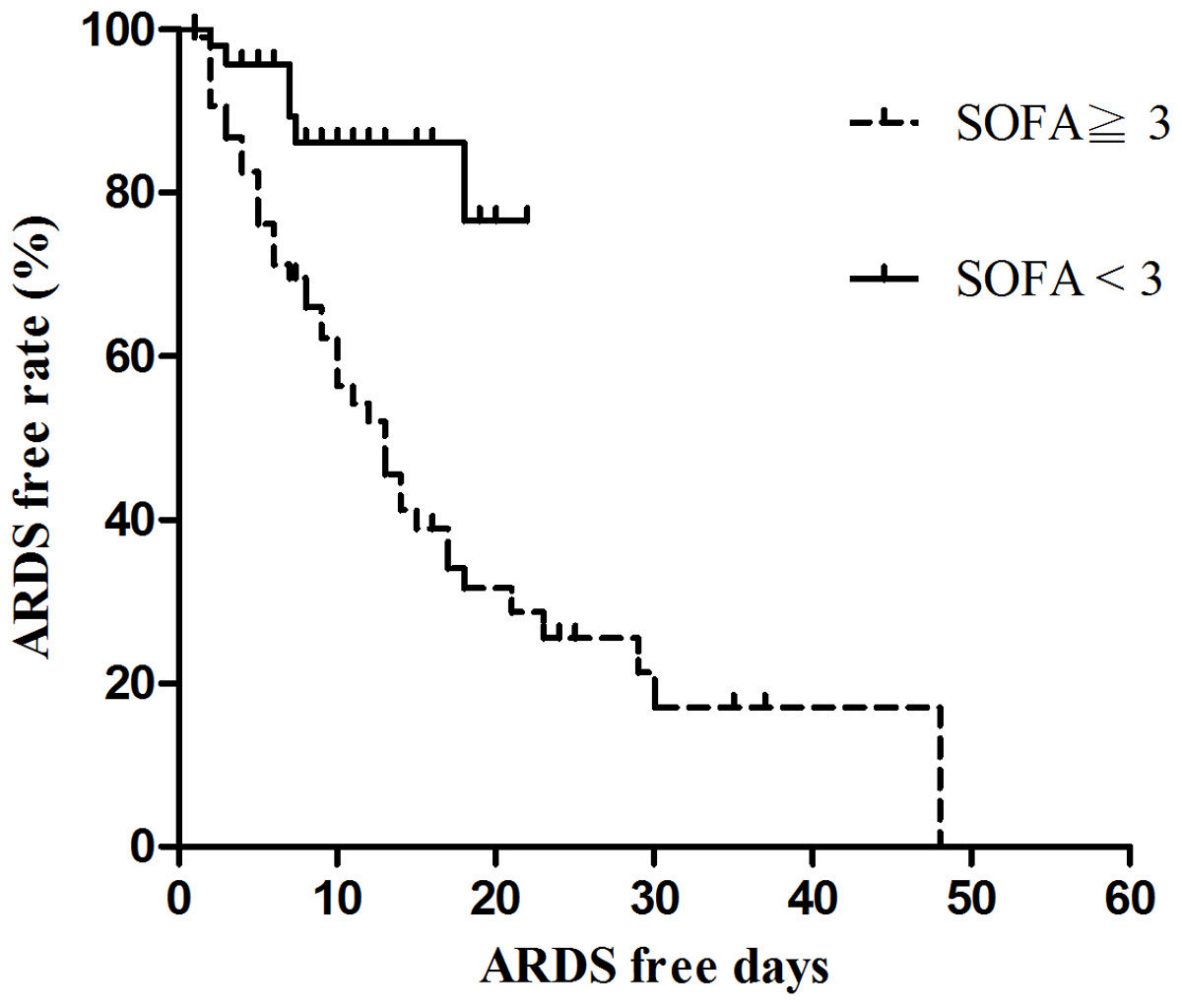
Cumulative ARDS free rates based on SOFA 48-h score.

Univariate Cox regression identified several clinical variables that were significantly associated with ARDS (Table 5). Multivariate Cox regression analyses indicated that blood paraquat concentrations (*P*<0.001), SOFA_48-h_ scores (*P*=0.001), and steroid and cyclophosphamide pulse therapies (*P*=0.024) were independent predictors of ARDS. Sensitivity, specificity, and overall correctness of SOFA_48-h_ score and blood paraquat concentration were calculated to determine their predictive values (Table 6). The cumulative survival rates differed significantly (*P*<0.001) between patients with SOFA_48-h_ scores <3 and SOFA_48-h_ scores ≥3 (Figure 1).

**Table 5:** Analysis of mortality using univariate and multivariate Cox regression models (n = 187).

	B	SE	Exp(B)	*P*
*Univariate*				
Time to hospital (hours)	-0.019	0.009	0.983 (0.964-0.998)	0.026*
Estimated ingestion amount (mL)	0.004	0.001	1.004 (1.001-1.007)	0.006**
Steroid and cyclophosphamide pulse therapies	-1.029	0.530	0.357 (0.127-0.996)	0.048*
Blood paraquat concentration (ppm)	0.156	0.026	1.169 (1.111-1.231)	<0.001***
eGFR (ml/min)	-0.018	0.006	0.982 (0.970-0.994)	0.004**
SOFA_48-h_	0.215	0.052	1.240 (1.120-1.372)	<0.001***
*Multivariate*				
Blood paraquat concentration (ppm)	0.135	.027	1.145 (1.086-1.208)	<0.001***
SOFA_48-h_	0.182	0.057	1.200 (1.074-1.341)	0.001**
Pulse therapy	-1.239	0.548	0.290 (0.099-0.849)	0.024*

Note: eGFR estimated glomerular filtration rate, SOFA Sequential Organ Failure Assessment, *P<0.05, **P<0.01, ***P<0.001

**Table 6:** Prediction of ARDS (n=187).

Predictive factors	Cutoff point	Youden index	Sensitivity %	Specificity %	Overall correctness %
Blood paraquat concentration (PPM)	1	0.312	61.4	69.8	54.0
SOFA_48-h_	3	0.542	95.8	58.4	67.6

SOFA: sequential organ failure assessment

## Discussion

In this study, we demonstrated that SOFA_48-h_ scores, blood paraquat concentrations, and steroid and cyclophosphamide pulse therapies were significant predictors for ARDS after paraquat poisoning. Many clinical parameters and scoring systems have previously been proposed as mortality predictors for patients with paraquat intoxication[2,19,20]. SOFA scores have been widely used in predicting outcomes for ARDS patients in general medical and/or surgical intensive care units[21-26]. Nevertheless, SOFA scores have never been utilized to predict ARDS complications in paraquat cases. Thus, this investigation appears to be the first report in the literature to demonstrate that SOFA scores >3 are predictors for ARDS with a sensitivity of 95.8%, specificity of 58.4%, and overall correctness of 67.6% (Table 6 and Figure 1). 

The SOFA system is a simple, easily performed, inexpensive, and reproducible scoring method. It is suitable for use in ordinary wards where most patients with paraquat intoxication are admitted. Most importantly, SOFA scores also include parameters of major target organs such as the lungs, liver, and kidneys. The average time of ARDS development was 10.04 ± 9.01 days in this study (Table 3). Hence, the SOFA_48-h_ score can predict ARDS earlier and thus can enable intervention such as steroid or cyclophosphamide pulse therapies[4-8] to be performed as soon as possible. As previously mentioned, the SOFA scores include indicators of liver damage, which is also a major sequel of paraquat toxicity[12] and patients with severe liver damage might suffer from a life-threatening hepatopulmonary syndrome, which is characterized by defects in oxygenation caused by pulmonary abnormalities associated with liver damage[13,14].

Our report and other previous reports[2,27] revealed that the blood paraquat concentration was the most consistent significant predictor of mortality after intoxication. In this study, we further demonstrated that the blood paraquat concentration was also a significant predictor of ARDS in paraquat poisoning. However, serum paraquat concentrations decreased rapidly within the first few hours after ingestion, and the time intervals between ingestion and serum paraquat measurements varied from patient to patient. Therefore, the relationship between ARDS and serum paraquat concentrations might not be reliable. Furthermore, the AUROC, sensitivity, specificity, and overall correctness of the blood paraquat concentration in predicting ARDS complications were inferior to the SOFA_48-h_ score (Table 4 and Table 6). As shown in Table 4, the AUROC analysis showed that SOFA_48-h_ scores (*P*<0.001) had a better discriminatory power than blood paraquat concentrations (*P*=0.01) for predicting ARDS.

Based on the findings of 3 randomized controlled trials of moderate to severely poisoned patients[3,28,29], glucocorticoids with cyclophosphamide in addition to standard care may be a beneficial treatment for patients with paraquat-induced lung fibrosis. Similarly, in this study, we found that patients with paraquat intoxication treated with steroids with cyclophosphamide pulse therapy experienced lower ARDS complication rates (odds ratio 0.290, 95% confidence interval 0.099–0.849, *P*=0.024, Table 5) compared to those in patients who were treated without these treatments. Unless contraindicated, it is suggested that all paraquat cases be aggressively treated with steroids and cyclophosphamide pulse therapies as early as possible[30].

Pneumomediastinum following paraquat poisoning is uncommon according to this observation but comprised 38.45% and 18.75% of cases in other surveys[31,32]. The development of pneumomediastinum following paraquat ingestion could be explained by the Macklin effect[33]. Paraquat toxicity causes acute inflammation of the lung, which results in necrotizing lung parenchyma, and late pulmonary fibrosis. Therefore, the lung will become stiff, predisposing these patients to barotrauma. Free air might track from ruptured alveoli along the peribronchial vascular sheaths toward the hilum of the lung; from there, it might extend proximally to the mediastinum[33]. Pneumomediastinum in paraquat poisoning often designates a grave prognosis, with a mortality rate[32] of almost 100%. In this study, 7 patients (3.7%) suffered from pneumomediastinum, of which 5 patients had ARDS and 2 patients did not (*P*<0.001). As expected, 6 of the 7 patients with pneumomediastinum died (mortality rate = 85.7%) despite an intensive detoxification protocol. 

As shown in Table 5, time to hospital after paraquat poisoning was a significant negative predictor of ARDS using a univariate Cox regression model (*P*=0.026), and the relationship did not exist after multivariate analysis (*P*=0.093). Furthermore, patients with ARDS arrived at the hospital sooner than those without ARDS, although this did not reach a significant level (*P*=0.438, Table 3). The increased ARDS that was observed for patients who presented more quickly to the hospital might be attributable to the fact that patients with more severe symptoms tended to seek medical attention faster.

A chi-square analysis demonstrated that the ARDS patients suffered from higher AKIN stages than those in the non-ARDS patients (*P*=0.009, Table 3). Our previous studies[2,12] revealed that paraquat toxicity caused substantial acute kidney injury[22]. Moreover, the ARDS patients represented a population with a high risk of acute kidney injury. There is a cross talk between renal and pulmonary damage[15]. Liu et al. [34] reported that clinical patients with acute lung injury constantly have increased plasma biomarkers such as plasminogen activator inhibitor-1, interleukin-6, and soluble tumor necrosis factor receptors after acute kidney injury, suggesting that disordered coagulation, inflammation, and neutrophil-endothelial interactions play important roles in the pathogenesis of acute kidney injury. An experimental study[35] revealed an elevation of macrophage-derived inflammatory products and an increase in pulmonary vascular permeability after isolated renal ischemia/reperfusion injury in rats. Therefore, acute kidney injury following paraquat poisoning may result from the toxicity of paraquat itself or as a renal complication of acute lung injury.

## Conclusions

In conclusion, the findings of this investigation indicated that SOFA_48-h_ scores, blood paraquat concentrations, and steroid and cyclophosphamide pulse therapies are independently associated with ARDS complications after paraquat intoxication. Furthermore, the analysis suggested that the SOFA_48-h_ score has the best discriminative power for predicting ARDS development. Further studies are warranted.
